# deepGBLUP: joint deep learning networks and GBLUP framework for accurate genomic prediction of complex traits in Korean native cattle

**DOI:** 10.1186/s12711-023-00825-y

**Published:** 2023-07-31

**Authors:** Hyo-Jun Lee, Jun Heon Lee, Cedric Gondro, Yeong Jun Koh, Seung Hwan Lee

**Affiliations:** 1 Department of Bio-AI Convergence, Chungnam National University, 305-764 Daejeon, Korea; 2 Division of Animal and Dairy Science, Chungnam National University, 305-764 Daejeon, Korea; 3 Department of Computer Science and Engineering, Chungnam National University, 305-764 Daejeon, Korea; 4 Department of Animal Science, Michigan State University, East Lansing, MI USA

## Abstract

**Background:**

Genomic prediction has become widespread as a valuable tool to estimate genetic merit in animal and plant breeding. Here we develop a novel genomic prediction algorithm, called deepGBLUP, which integrates deep learning networks and a genomic best linear unbiased prediction (GBLUP) framework. The deep learning networks assign marker effects using locally-connected layers and subsequently use them to estimate an initial genomic value through fully-connected layers. The GBLUP framework estimates three genomic values (additive, dominance, and epistasis) by leveraging respective genetic relationship matrices. Finally, deepGBLUP predicts a final genomic value by summing all the estimated genomic values.

**Results:**

We compared the proposed deepGBLUP with the conventional GBLUP and Bayesian methods. Extensive experiments demonstrate that the proposed deepGBLUP yields state-of-the-art performance on Korean native cattle data across diverse traits, marker densities, and training sizes. In addition, they show that the proposed deepGBLUP can outperform the previous methods on simulated data across various heritabilities and quantitative trait loci (QTL) effects.

**Conclusions:**

We introduced a novel genomic prediction algorithm, deepGBLUP, which successfully integrates deep learning networks and GBLUP framework. Through comprehensive evaluations on the Korean native cattle data and simulated data, deepGBLUP consistently achieved superior performance across various traits, marker densities, training sizes, heritabilities, and QTL effects. Therefore, deepGBLUP is an efficient method to estimate an accurate genomic value. The source code and manual for deepGBLUP are available at https://github.com/gywns6287/deepGBLUP.

**Supplementary Information:**

The online version contains supplementary material available at 10.1186/s12711-023-00825-y.

## Background

The use of DNA marker information for the prediction of genetic merit in animal and plant breeding and susceptibility to disease in human medicine has become widespread. Genomic prediction has primarily utilized many thousands of DNA markers, most commonly single nucleotide polymorphisms (SNPs), that cover the entire genome to predict the genetic merit and phenotypes of individuals. In humans, genomic prediction has been widely used to predict disease risk and highly polygenic complex human traits [1, 2]. In agriculture, genomic prediction is used to estimate a genomic value (GV), which is then used to make selection decisions in a breeding population.

Genomic best linear unbiased prediction (GBLUP) is one of the most commonly used statistical models for genomic prediction [3]. It adopts a mixed model approach that uses a genomic relationship matrix (GRM) built from genotypes instead of a traditional pedigree-based relationship matrix. Even though this method showed state-of-the-art performance in many populations, it still has some limitations. First, it approximates a traditional infinitesimal model, which assumes an equal genetic variance for all SNPs. To resolve this limitation, Bayesian models [4, 5] assume that some SNPs have zero effects, whereas others have small to moderate effects. However, these methods require unknown parameters to be calculated by multiple iterations, which is time-consuming. Fragomeni et al. [6] and Wang et al. [7] derived the optimal weights of SNPs to allow unequal variances for each SNP in the GBLUP equation, but they only brought a negligible improvement in simulation data. Ren et al. [8] developed a weighting method to construct a weighted GRM, but it required additional priorities to estimate SNP effects. Furthermore, the conventional GBLUP method only accounts for additive marker effects due to its reliance on a linear model. To interfuse non-linearity effects into GBLUP, some studies focused on deriving GRM with dominance effects [9, 10] and epistatic interactions [11, 12]. However, the line of research that directly leverages the non-linearity to GV estimation was less studied.

Deep learning is a good alternative method to solve these problems. Recent advances in deep neural networks have outperformed the state-of-the-art in various fields, such as computer vision, machine translation, autonomous driving, and audio recognition [13–17]. In particular, the use of local information has led to these successes. Convolutional neural network (CNN), which is the most common structure for computer vision, constitutes a weights-shared filter operation for the adjacent region of an input image [18]. Recurrent neural network (RNN) has been commonly used in sequence-to-sequence problems, such as speech recognition or natural language processing [14]. It takes information from previous sequence positions to extract information from a current sequence position. These two networks hypothesize that adjacent regions with similar patterns could provide shared features between them. More recently, the transformer [16], an advanced deep learning method, has achieved superior performance in the computer vision [17] and the natural language processing [19]. It also exploits a relative position to extract informative features from input data.

The local information can also be exploited in genomic prediction. The general concept of genomic prediction relies on the linkage disequilibrium (LD) between genetic markers and the unknown quantitative trait loci (QTL). With high-density SNP panels, the markers co-segregate with the causal mutations, allowing the effects of causal variants to be indirectly estimated through adjacent markers [5, 20]. Therefore, it is essential to carefully use the information of adjacent markers for accurate genomic prediction. However, previous deep learning networks, such as CNN or RNN, are not suitable to estimate adjacent marker effects, since they assign marker effects based primarily on sequence patterns. In SNP array data, adjacent markers often lose a functional relation (e.g. protein coding) due to varying distances between them. In other words, adjacent SNPs with the same pattern but located in different loci can have different functional effects from each other. Practically, simple fully-connected networks that do not use local information usually perform better than other local-based networks in genomic prediction [21, 22]. In this regard, a new local-based network is needed to capture the effects of adjacent markers considering their distinct loci.

There have been many attempts to leverage deep learning networks for genomic prediction. Zingaretti et al. [23] explored CNN for genomic prediction of polyploid outcrossing species. Montesinos-López et al. [24] used various deep learning architectures for multi-environment genomic predictions of complex traits in plants. Pook et al. [25] applied locally-connected layers on simulated maize and real Arabidopsis data. However, as these methods cannot achieve a sufficient accuracy even with more complex parameters than conventional methods, they quickly reach the limit to their uses in real-world applications.

**Fig. 1:**
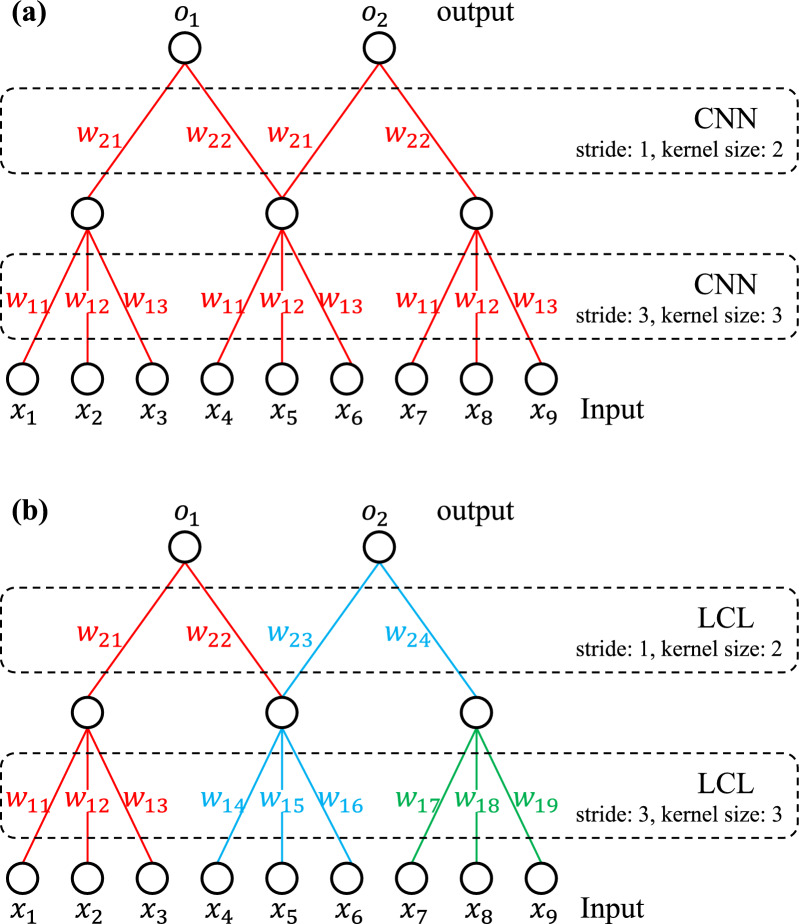
Example of a convolution neural network and a locally connected layer. **a** Convolution neural networks (CNN); **b** Locally-connected layer (LCL). Different colors mean different weight sets

To this end, we propose a novel algorithm, which is a joint deep learning networks and GBLUP framework (deepGBLUP) for accurate genomic prediction. Given the SNP sequence data, the proposed deepGBLUP first extracts the effects of adjacent markers using a locally connected layer (LCL). Figure 1 compares LCL with the common CNN. LCL works similarly to CNN, except that weights in each filter are unshared. Therefore, distinct weight sets are used for adjacent markers located at different loci. Then, deepGBLUP estimates an initial GV from the effects of adjacent markers through a fully-connected layer. However, this initial GV lacks a concrete genetic relationship, which may generate un-reliable results as in the previous studies [23–25]. The genetic relationship between training and test individuals is crucial for genomic prediction. To address this, we leveraged a well-modified GBLUP framework that can utilize genomic relationships (i.e. GRM) for a GV estimation. The proposed GBLUP framework estimates additive, dominance, and epistatic GV using three types of GRM. The implementation details about the GBLUP framework are available in the Methods section. Then, the proposed deepGBLUP estimates a final GV by summing the initial, additive, dominance, and epistatic GV. We evaluated deepGBLUP using a Korean native cattle dataset that covers diverse marker densities, training sizes, and traits. In addition, we validated its performance on simulated data involving various ranges of heritabilities and QTL effects.

## Methods

### Korean native cattle dataset

The Korean native cattle population used in this study included 10,000 individuals (animals were born between 2010 and 2017, and samples were collected between 2013 and 2019) with phenotypic measurements for carcass weight (CWT/kg), eye-muscle area (EMA/cm$^2$), backfat thickness (BF/mm), and marbling score (MS). CWT was measured by scales on beef production rails in the slaughterhouse. BF, EMA, and MS at the junction between the 12th and 13th ribs were manually measured by human experts after a 24-h chill.

Genomic DNA of the animals was extracted from *longissimus-thoracis* muscle samples using a DNeasy Blood and Tissue Kit (Qiagen, Valencia, CA). In total, 10,000 samples were genotyped using the Illumina Bovine SNP50 BeadChip. SNP quality control was performed using the PLINK1.9 software [26] based on the following filtering criteria: SNPs with a minor allele frequency < 0.001, a call rate < 0.1 and those located on the sex chromosomes were removed, i.e. 1853 SNPs, and the post-filter missing rate was 0.6% of the genotypes. These missing SNPs were then imputed with Eagle v2.4 [27]. Finally, 44,314 SNPs were used in the study, which are defined as the 50K set. Furthermore, we selected 10K, 5K, and 1K evenly distributed markers from the 50K set to evaluate deepGBLUP performance across marker densities.

All experimental procedures were approved by the National Institute of Animal Science (NIAS) in the Rural Development Administration (RDA) of South Korea, and all samples were taken under public animal health and welfare guidelines.

### Simulated dataset

We used the Qmsim1.10 [28] software to simulate 10,000 individual genotypes. In the simulated data, 49,980 SNPs were uniformly distributed across the 29 chromosomes. According to the Korean breeding program [29], the Korean cattle population has been established, starting with a few outstanding individuals. To imitate the mutations and LD structures of the Korean native cattle, a historical population was simulated with 200 individuals (100 males and 100 females) for 1000 generations and maintaining constant population size by random mating. Then the population size was gradually increased to 10,000 individuals (5000 males and 5000 females) for 20 additional generations (1001th–1020th). We used these simulated genotypes as a basis and modeled 21 phenotypes with three heritabilities *h*^2^ (0.5, 0.3, and 0.1) and seven QTL effect combinations, including additive (a), dominance (d), epistasis (e), additive + dominance (a+d), additive + epistasis (a + e), dominance + epistasis (d + e), and additive + dominance + epistasis (a + d + e).

To model each phenotype, we first drew the polygenic effects of all SNPs from a ${\mathcal {N}}(0,1)$ distribution. The weighted sum of the SNPs by their polygenic effects was used as an individual’s polygenic effect, where SNPs were coded as 0, 1, and 2 for the reference homozygote, heterozygote, and alternate homozygote genotype, respectively.

To simulate QTL effects, we randomly selected 1000 additive, 1000 dominance, and 1000 epistasis QTL from the 49,980 SNP set. It should be noted that each QTL was selected from loci that were free from any other QTL. The additive effects (a) were computed by the weighted sum of 1000 additive QTL by their effects from a ${\mathcal {N}}(0,1)$ distribution. For the dominance effects (d), we re-coded the genotypes of 1000 dominance QTL to 0, 1, and 1, resulting in an additive and a dominance effect of equal size. As with the additive effects, individual dominance was calculated by the weighted sum of dominance genotypes by their effects drawn from a ${\mathcal {N}}(0,1)$ distribution. To model the epistatic effects (e), we followed the simulation scheme in [11]. Specifically, one of the nine possible configurations of the 499,500 QTL pairs was randomly chosen to have a ${\mathcal {N}}(0,1)$ distributed effect. For instance, when the marker pair $x_c, x_l$ is drawn, only the configuration $(x_c=0, x_l=2)$ has an effect. We calculated the individual epistasis (e) by summing the total epistasis effect of QTL pairs.

We standardized the variance of each effect to restrain them into the target heritabilities (0.5, 0.3, and 0.1). Let $\sigma ^2_p$ be a phenotype variance, which was set to 100 in this study. We first drew the residuals of the individuals from ${\mathcal {N}}(0,\sqrt{(1-h^2)\sigma ^2_p})$. Then the variances of the polygenic effects were standardized to $\frac{7}{10} h^2\sigma ^2_p$, while the variances of the additive (a), dominance (d), and epistatic (e) effects were each standardized to $\frac{1}{10} h^2\sigma ^2_p$. Note also that all 21 phenotypes included polygenic effects and residuals with different heritabilities and different combinations of a, d, and e.

### Joint deep learning networks and GBLUP framework (deepGBLUP)

**Fig. 2:**
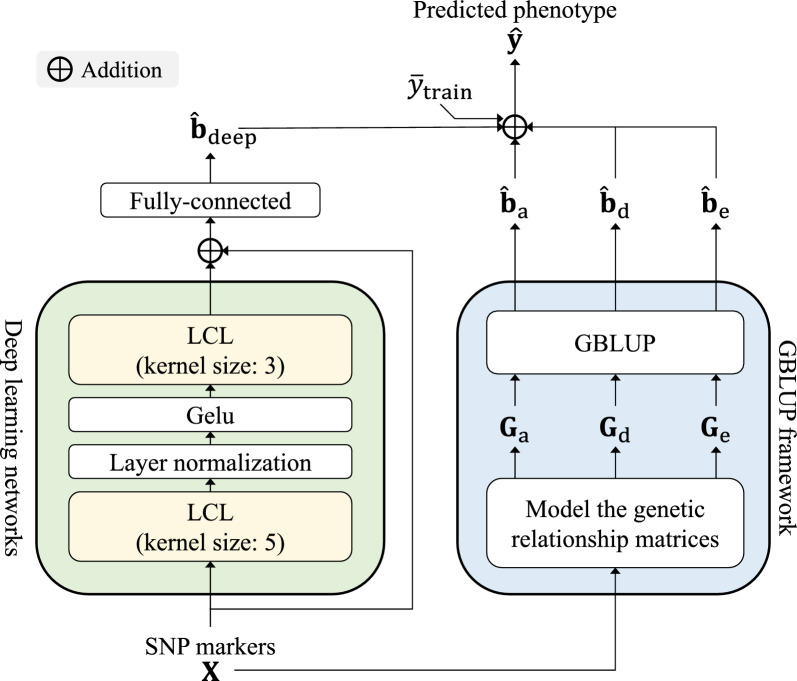
Overview of the proposed deepGBLUP

In this study, we propose a novel genomic prediction method, which integrates deep learning networks and a GBLUP framework (deepGBLUP). The deep learning networks extract the effects of adjacent markers using locally-connected layers and subsequently use them to estimate an initial GV through fully-connected layers. The GBLUP framework estimates three types of GV (additive, dominance, and epistasis) by leveraging the respective genomic relationship matrices. We addressed individuals with known and unknown phenotypes as training and test individuals, respectively. Then the goal of deepGBLUP is to predict phenotypes of the test individuals from an input SNP sequence and known phenotypes of training individuals. Figure 2 illustrates an overview of the proposed deepGBLUP.

As in Fig. 2, we decomposed *n* individuals’ phenotype ${\textbf{y}} \in \mathbb {R}^{n}$ into five components: mean term $\mu$, initial GV ${\textbf{b}}_\text {deep} \in \mathbb {R}^{n}$, additive GV ${\textbf{b}}_\text {a} \in \mathbb {R}^{n}$, dominance GV ${\textbf{b}}_\text {d} \in \mathbb {R}^{n}$, epistatic GV ${\textbf{b}}_\text {e} \in \mathbb {R}^{n}$, and a residual vector ${\textbf{r}} \in \mathbb {R}^{n}$:

1 $$\begin{aligned} {\textbf{y}} = \mu + {\textbf{b}}_\text {deep} + {\textbf{b}}_\text {a} + {\textbf{b}}_\text {d} + {\textbf{b}}_\text {e} + {\textbf{r}}. \end{aligned}$$

As the mean term $\mu$ can be calculated from the training individuals’ known phenotypes, the genomic prediction of deepGBLUP can be summarized to estimate the four different GV, ${\textbf{b}}_\text {deep}$, ${\textbf{b}}_\text {a}$, ${\textbf{b}}_\text {d}$, and ${\textbf{b}}_\text {e}$. Specifically, the proposed deepGBLUP estimates $\hat{{\textbf{b}}}_\text {a}$, $\hat{{\textbf{b}}}_\text {d}$ and $\hat{{\textbf{b}}}_\text {e}$ using the GBLUP framework, while $\hat{{\textbf{b}}}_\text {deep}$ is estimated using the deep learning networks as shown in Fig. 2.

#### GBLUP framework

The commonly used GBLUP equation [3] to predict a genomic value $\hat{{\textbf{b}}}$ is defined as:

2 $$\begin{aligned} \text {GBLUP}({\textbf{G}},{\textbf{y}}_\text {train}) = \hat{{\textbf{b}}}^T = [{\textbf{Z}}^T{\textbf{Z}} + \lambda {\textbf{G}}^{-1}]^{-1}{\textbf{Z}}^T({\textbf{y}}_\text {train} - {\bar{y}}_\text {train})^T, \end{aligned}$$

where ${\textbf{G}} \in \mathbb {R}^{n \times n}$ is a genomic relationship matrix between all *n* individuals, ${\textbf{y}}_\text {train} \in \mathbb {R}^{n_\text {train}}$ is a known phenotype vector of the $n_\text {train}$ train individuals, and ${\bar{y}}$ is a mean of ${\textbf{y}}_\text {train}$. ${\textbf{Z}} \in \{0, 1\}^{n_\text {train} \times n}$ is an incidence matrix for which the diagonals are set to 1 for the training individual columns and the others are 0. $\lambda$ is a normalizing scalar, which is commonly set to $(1-h^2)/h^2$ in the regular GBLUP. Note that the regular GBLUP can be classified into additive [3], dominance [9], and epistasis [30]-GBLUP depending on which matrix is used to replace ${\textbf{G}}$.

The proposed deepGBLUP estimates additive GV $\hat{{\textbf{b}}}_\text {a}$, dominance GV $\hat{{\textbf{b}}}_\text {d}$, and epistatic GV $\hat{{\textbf{b}}}_\text {e}$, using Eq. (2) with an additive relationship matrix ${\textbf{G}}_\text {a} \in \mathbb {R}^{n \times n}$, a dominance relationship matrix ${\textbf{G}}_\text {d} \in \mathbb {R}^{n \times n}$, and an epistasis relationship matrix ${\textbf{G}}_\text {e} \in \mathbb {R}^{n \times n}$, respectively. The genotype data of all *n* individuals can be written as a matrix ${\textbf{X}} \in \{0,1,2\}^{n \times p }$, for which the column dimension *p* is the number of SNPs. Each element 0, 1, and 2 in ${\textbf{X}}$ refers to the reference homozygote, heterozygote, and alternate homozygote genotype, respectively. We calculated the additive relationship matrix ${\textbf{G}}_\text {a}$ following [3]:

3 $$\begin{aligned}&\tilde{{\textbf{X}}} = {\textbf{X}} - 2{\textbf{P}}, \end{aligned}$$

4 $$\begin{aligned}&{\textbf{G}}_\text {a} = \frac{\tilde{{\textbf{X}}}\tilde{{\textbf{X}}}^T}{2\sum {p_i(1-p_i)}}, \end{aligned}$$

where $p_i$ is the allele frequency of the *i*th marker and ${\textbf{P}} \in \mathbb {R}^{n \times p}$ is an extended matrix, in which the rows are an allele frequency vector ${\textbf{p}} \in \mathbb {R}^{p}$.

We constructed the dominance relationship matrix ${\textbf{G}}_\text {d}$ by [9]. Under the assumption of Hardy-Weinberg equilibrium, a dominance value of the *i*th marker can be expressed as $-2p_i^2$, $2p_i(1-p_i)$, and $-2(1-p_i)^2$ for the reference homozygote, heterozygote, and alternate homozygote, respectively. Then the dominance values of all individuals can be written as a matrix ${\textbf{D}} \in \mathbb {R}^{n \times p}$. We computed the dominance relationship matrix ${\textbf{G}}_\text {d}$ by:

5 $$\begin{aligned} {\textbf{G}}_\text {d} = \frac{{\textbf{D}}{\textbf{D}}^T}{4\sum {p_i^2(1-p_i)^2}}. \end{aligned}$$

With a definition of the multivariate Gaussian distribution, the epistasis relationship matrix ${\textbf{G}}_\text {e}$ can be derived by [30]:

6 $$\begin{aligned}&\tilde{{\textbf{G}}}_\text {e} = 0.5({\textbf{M}}{\textbf{M}}^T \circ {\textbf{M}}{\textbf{M}}^T) - 0.5({\textbf{M}} \circ {\textbf{M}})({\textbf{M}} \circ {\textbf{M}})^T, \end{aligned}$$

7 $$\begin{aligned}&{\textbf{G}}_\text {e} = \frac{\tilde{{\textbf{G}}}_\text {e}}{\text {Tr}(\tilde{{\textbf{G}}}_\text {e})/n}, \end{aligned}$$

where ${\textbf{M}} \in \{-1, 0, 1\}^{n \times p}$ is a centered genotype ${\textbf{X}}-1$, $\circ$ is the Hadamard product, and $\text {Tr}(\cdot )$ is the trace operation that is a sum of matrix diagonals. For the detailed derivation of this equation, please see [30].

**Fig. 3:**
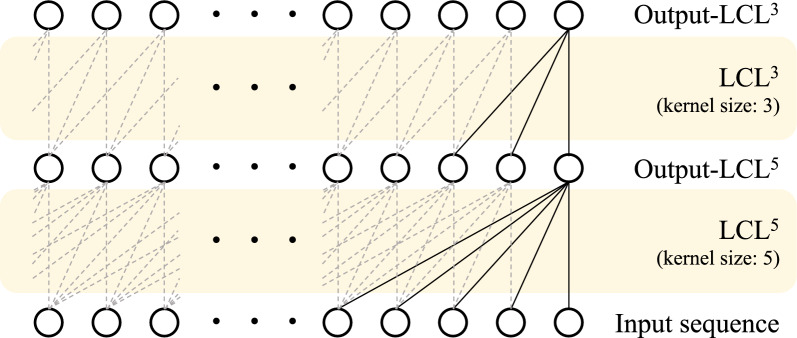
Detailed description of the proposed locally-connected layer. LCL$^{k}$ is a locally connected layer with *k* kernel size

#### Deep learning networks

Figure 3 illustrates the proposed locally connected layer (LCL). It recursively aggregates *k* adjacent SNPs across the whole sequence with one stride. Let ${\textbf{t}} \in \mathbb {R}^{p}$ and ${\textbf{o}} \in \mathbb {R}^{p}$ be the input and output sequence of LCL. The proposed LCL calculates the *m*th value $o_{m}$ of the output sequence ${\textbf{o}}$ as follow:

8 $$\begin{aligned} o_{m} = \sum ^{k-1}_{j=0}w_{m,(j+1)}t_{(m-j)}, \end{aligned}$$

where *k* is the kernel size and $w_{m,j}$ is the *j*th kernel weight for the *m*th output in trainable weight matrix ${\textbf{W}} \in \mathbb {R}^{p \times k}$. Then, the LCL operation with the kernel size *k* can be written as:

9 $$\begin{aligned} &\text {LCL}^k({\textbf{t}},{\textbf{W}}) = {\textbf{o}} \\&\quad = \left[ \sum ^{k-1}_{j=0}w_{1(j+1)}t_{(1-j)}, \cdots , \sum ^{k-1}_{j=0}w_{m(j+1)}t_{(m-j)}, \cdots , \sum ^{k-1}_{j=0}w_{p(j+1)}i_{(p-j)} \right] . \end{aligned}$$

Note that LCL cannot be performed when $m \le j$, since there must be no value $t_{(m-j)}$ at a negative position. Thus, LCL replaces $t_{(m-j)}$ as zero value when $m \le j$. To extract high-level features of input SNPs, deepGBLUP adopts sequential LCL as shown in Fig. 2. Let ${\textbf{x}}_i$ be the *i*th individual’s SNP sequence. The proposed deepGBLUP first extracts the temporal marker effects of the *i*th individual $\tilde{{\textbf{e}}}_i \in \mathbb {R}^{p}$ through LCL$^5(\cdot )$:

10 $$\begin{aligned} \tilde{{\textbf{e}}}_i = \text {GeLU}(\text {LN}(\text {LCL}^5({\textbf{x}}_i,{\textbf{W}}_{\tilde{\text {e}}}))), \end{aligned}$$

where LN($\cdot$) is a layer normalization [31], GeLU($\cdot$) is a GELU non-linearity [32], and ${\textbf{W}}_{\tilde{\text {e}}} \in \mathbb {R}^{p \times k}$ is a trainable weight of $\text {LCL}^5$. Then, the final marker effects ${\textbf{e}}_i \in \mathbb {R}^{p}$ are calculated by:

11 $$\begin{aligned} {\textbf{e}}_i = \text {LCL}^3(\tilde{{\textbf{e}}}_i,{\textbf{W}}_{\text {e}}), \end{aligned}$$

where ${\textbf{W}}_{\text {e}} \in \mathbb {R}^{p \times k}$ is a trainable weight of $\text {LCL}^3$. To ensure the reusability of input sequences, deepGBLUP adds marker effects ${\textbf{e}}_i$ to input SNPs $\tilde{{\textbf{x}}}_i = {\textbf{x}}_i + {\textbf{e}}_i$. Then, the effect-interfused SNPs of all *n* individuals can be presented by a matrix $\tilde{{\textbf{X}}} \in \mathbb {R}^{n \times p}$. Finally, deepGBLUP estimates an initial GV $\hat{{\textbf{b}}}_\text {deep}$ from ${\tilde{{\textbf{X}}}}$ through a fully-connected layer (FCL):

12 $$\begin{aligned} \hat{{\textbf{b}}}_\text {deep}^T = \text {FCL}({\tilde{{\textbf{X}}}},{\textbf{W}}_\text {b}) = {\tilde{{\textbf{X}}}}{\textbf{W}}_\text {b}, \end{aligned}$$

where ${\textbf{W}}_\text {b} \in \mathbb {R}^{p \times 1}$ is a trainable weight of FCL. Then, it computes the predicted phenotype $\hat{{\textbf{y}}} \in \mathbb {R}^n$ of all *n* individuals by $\hat{{\textbf{y}}} = {\bar{y}}_\text {train} + \hat{{\textbf{b}}}_\text {deep} + \hat{{\textbf{b}}}_\text {a} + \hat{{\textbf{b}}}_\text {d} + \hat{{\textbf{b}}}_\text {e}$.

#### Loss function and implementation details

For training deepGBLUP, we employed L1-loss between observed and predicted phenotypes of training individuals:

13 $$\begin{aligned} L = \frac{1}{n_\text {train}}\sum _{i=1}^{n_\text {train}}| y^{(i)}_\text {train} - {\hat{y}}^{(i)}_\text {train}|, \end{aligned}$$

where $y^{(i)}_\text {train}$ and ${\hat{y}}^{(i)}_\text {train}$ are the *i*th value of ${\textbf{y}}_\text {train}$ and $\hat{{\textbf{y}}}_\text {train}$, respectively. Thus, the proposed deepGBLUP iteratively optimized the trainable weights set ${\mathcal {W}} = \{{\textbf{W}}_{\tilde{\text {e}}}, {\textbf{W}}_\text {e}, {\textbf{W}}_\text {b} \}$ to minimize *L* during the training process. We used AdamW [33] for the parameter optimization.

To evaluate deepGBLUP performance, we measured the Pearson correlation coefficient between $\hat{{\textbf{y}}}_\text {test}$ and ${\textbf{y}}_\text {test}$, divided by the square root of heritability, i.e. $\text {cor}(\hat{{\textbf{y}}}_\text {test}, {\textbf{y}}_\text {test})/h$. We defined this as predictive ability in this study. We estimated the heritability using an average information-restricted maximum likelihood [34] in the AIREMLF90 software [35]. By this method, the heritabilities of each trait were estimated to 0.392, 0.378, 0.366, and 0.479 for CWT, BF, EMA, and MS, respectively.

We conducted comparative analyses for the proposed deepGBLUP with state-of-the-art genomic prediction algorithms, including GBLUP [3], dominance GBLUP (DGBLUP [9]) and epistasis GBLUP (EGBLUP [30]), BayesA [4], BayesB [4], and BayesC [4]. GBLUP yields an additive GV as an output, while DGBLUP and EGBLUP incorporate dominance+additive GV and epistatic+additive GV, respectively. We implemented all Bayesian models using the BGLR [36] package in R program language. We also used a 10-fold cross-validation scheme to evaluate model performance. All individuals were divided into 10 groups of equal size. Nine of these groups were used as the training individuals and the other group was used as the test individuals in each cross-validation. The means and standard errors of predictive abilities, aggregated over the 10-fold tests, are reported in this study as performance metrics.

## Results

### Model performance on the Korean native cattle data

We determined a learning rate and an epoch using a validation stage. Specifically, we selected 10% of the training individuals as validation individuals. Then, we trained deepGBLUP using the other 90% of the training individuals and evaluated its performance using the validation individuals. Finally, we selected a learning rate and an epoch, which achieved the best performance on the validation individuals. The determined learning rates and epochs for each trait across marker densities and training sizes are in Table 1. The training and test of deepGBLUP were conducted on an RTX A6000 GPU. With an efficient GPU device, deepGBLUP is able to predict the phenotypes of the individuals with a reasonable computing time as shown in Table 2.

**Table 1:** Determined epochs and learning rates (lr) to train deepGBLUP

Density	Train size	CWT		BF		EMA		MS	
		lr	epoch	lr	epoch	lr	epoch	lr	epoch
50K	9000	0.001	7	0.0001	9	0.001	3	0.0001	8
10K	9000	0.001	9	0.0001	12	0.001	4	0.0001	12
5K	9000	0.001	9	0.0001	13	0.001	5	0.001	2
1K	9000	0.001	23	0.0001	30	0.001	10	0.001	11
50K	5000	0.001	8	0.0001	15	0.001	4	0.0001	10
50K	2500	0.001	10	0.0001	9	0.001	5	0.0001	10
50K	1000	0.001	11	0.0001	4	0.001	6	0.0001	11

**Table 2:** Required times for training and test of deepGBLUP across marker densities and train sizes

Density	Train size	Training time (s)	Test time (s)
50K	9000	3.24	1.36
10K	9000	1.07	1.14
5K	9000	0.94	1.13
1K	9000	0.85	1.12
50K	5000	1.81	0.53
50K	2500	0.9	0.24
50K	1000	0.36	0.17

We recorded the average time of four traits (CWT, BF, EMA, and MS). Training time means a processing time for 1 epoch

#### Across marker density

Table 3 compares the proposed deepGBLUP with the other genomic prediction methods on the Korean native cattle dataset across various traits and marker densities. Notably, deepGBLUP demonstrates superior performance in all settings without exception. Even though Bayesian methods outperform the GBLUP methods, deepGBLUP exhibits a higher accuracy than Bayesian methods in all scenarios. These findings suggest that the deep learning networks can effectively complement the estimation results of the GBLUP methods.

**Table 3:** Performance comparison of deepGBLUP with the other genomic prediction methods on the Korean native cattle dataset across different traits and marker densities

Density	Method	CWT	BF	EMA	MS
50K	GBLUP	0.729 ± 0.015	0.647 ± 0.009	0.726 ± 0.017	0.670 ± 0.014
	DGBLUP	0.731 ± 0.016	0.639 ± 0.01	0.729 ± 0.017	0.668 ± 0.013
	EGBLUP	0.724 ± 0.016	0.641 ± 0.01	0.721 ± 0.019	0.664 ± 0.014
	BayesA	0.730 ± 0.015	0.658 ± 0.009	0.720 ± 0.016	0.667 ± 0.014
	BayesB	0.746 ± 0.015	0.667 ± 0.009	0.723 ± 0.019	0.670 ± 0.013
	BayesC	0.737 ± 0.015	0.662 ± 0.01	0.726 ± 0.018	0.668 ± 0.014
	deepGBLUP	*0.752* ± *0.016*	*0.673* ± *0.009*	*0.746* ± *0.017*	*0.672* ± *0.012*
10K	GBLUP	0.676 ± 0.015	0.577 ± 0.008	0.678 ± 0.018	0.613 ± 0.011
	DGBLUP	0.675 ± 0.015	0.571 ± 0.009	0.678 ± 0.018	0.607 ± 0.01
	EGBLUP	0.684 ± 0.016	0.585 ± 0.009	0.684 ± 0.019	0.619 ± 0.012
	BayesA	0.700 ± 0.015	0.59 ± 0.008	0.682 ± 0.019	0.620 ± 0.011
	BayesB	0.695 ± 0.015	0.585 ± 0.007	0.675 ± 0.018	0.612 ± 0.012
	BayesC	0.689 ± 0.016	0.589 ± 0.008	0.681 ± 0.018	0.616 ± 0.012
	deepGBLUP	*0.713* ± *0.017*	*0.612* ± *0.008*	*0.705* ± *0.018*	*0.626* ± *0.012*
5K	GBLUP	0.638 ± 0.015	0.543 ± 0.01	0.631 ± 0.019	0.548 ± 0.011
	DGBLUP	0.632 ± 0.016	0.533 ± 0.011	0.633 ± 0.019	0.544 ± 0.011
	EGBLUP	0.653 ± 0.016	0.556 ± 0.011	0.646 ± 0.02	0.564 ± 0.012
	BayesA	0.668 ± 0.016	0.557 ± 0.009	0.650 ± 0.019	0.568 ± 0.013
	BayesB	0.658 ± 0.016	0.543 ± 0.008	0.643 ± 0.018	0.562 ± 0.013
	BayesC	0.655 ± 0.017	0.555 ± 0.008	0.647 ± 0.019	0.567 ± 0.013
	deepGBLUP	*0.681* ± *0.016*	*0.58* ± *0.01*	*0.672* ± *0.019*	*0.582* ± *0.011*
1K	GBLUP	0.535 ± 0.017	0.429 ± 0.014	0.537 ± 0.021	0.424 ± 0.013
	DGBLUP	0.519 ± 0.015	0.401 ± 0.012	0.529 ± 0.023	0.405 ± 0.014
	EGBLUP	0.552 ± 0.017	0.444 ± 0.014	0.555 ± 0.022	0.443 ± 0.014
	BayesA	0.568 ± 0.016	0.442 ± 0.014	0.557 ± 0.022	0.443 ± 0.014
	BayesB	0.564 ± 0.016	0.437 ± 0.012	0.556 ± 0.021	0.441 ± 0.013
	BayesC	0.551 ± 0.017	0.440 ± 0.013	0.552 ± 0.021	0.441 ± 0.014
	deepGBLUP	*0.581* ± *0.016*	*0.467* ± *0.014*	*0.584* ± *0.022*	*0.466* ± *0.013*

Each value in the cells are means and standard errors of the predictive abilities for 10-fold tests. We highlight the best results in *italic*

#### Across training size

Deep learning methods typically require a large amount of data to operate effectively [15, 17, 19]. To identify the amount of data necessary for deepGBLUP, we evaluated its performances with varying training sizes of 5000, 2500, and 1000. The training individuals were randomly sampled in each 10-fold to obtain the corresponding training size. Table 4 presents a comparison of the proposed deepGBLUP with the other genomic prediction methods on the Korean native cattle dataset across various traits and training sizes. Our findings indicate that GBLUP-based methods outperform Bayesian methods for smaller training sizes (2500 and 1000). On the other hand, the proposed deepGBLUP consistently achieves the best predictive ability across all training sizes. These results demonstrate that deepGLBUP can yield stable performance even with less training data.

**Table 4:** Performance comparison of deepGBLUP with the other genomic prediction methods on the Korean native cattle dataset across different traits and training sizes

Train size	Method	CWT	BF	EMA	MS
9000	GBLUP	0.729 ± 0.015	0.647 ± 0.009	0.726 ± 0.017	0.670 ± 0.014
	DGBLUP	0.731 ± 0.016	0.639 ± 0.01	0.729 ± 0.017	0.668 ± 0.013
	EGBLUP	0.724 ± 0.016	0.641 ± 0.01	0.721 ± 0.019	0.664 ± 0.014
	BayesA	0.730 ± 0.015	0.658 ± 0.009	0.720 ± 0.016	0.667 ± 0.014
	BayesB	0.746 ± 0.015	0.667 ± 0.009	0.723 ± 0.019	0.670 ± 0.013
	BayesC	0.737 ± 0.015	0.662 ± 0.01	0.726 ± 0.018	0.668 ± 0.014
	deepGBLUP	*0.752* ± *0.016*	*0.673* ± *0.009*	*0.746* ± *0.017*	*0.672* ± *0.012*
5000	GBLUP	0.682 ± 0.018	0.581 ± 0.009	0.679 ± 0.018	0.609 ± 0.012
	DGBLUP	0.684 ± 0.018	0.576 ± 0.009	0.683 ± 0.019	0.610 ± 0.012
	EGBLUP	0.678 ± 0.017	0.578 ± 0.01	0.676 ± 0.019	0.606 ± 0.013
	BayesA	0.678 ± 0.018	0.581 ± 0.008	0.664 ± 0.017	0.602 ± 0.012
	BayesB	0.697 ± 0.017	0.593 ± 0.009	0.677 ± 0.019	0.606 ± 0.012
	BayesC	0.684 ± 0.018	0.586 ± 0.009	0.673 ± 0.019	0.607 ± 0.012
	deepGBLUP	*0.712* ± *0.018*	*0.607* ± *0.009*	*0.702* ± *0.018*	*0.619* ± *0.011*
2500	GBLUP	0.631 ± 0.016	0.515 ± 0.011	0.627 ± 0.025	0.539 ± 0.01
	DGBLUP	0.634 ± 0.016	0.514 ± 0.012	0.628 ± 0.024	0.539 ± 0.01
	EGBLUP	0.629 ± 0.016	0.514 ± 0.012	0.625 ± 0.025	0.538 ± 0.01
	BayesA	0.612 ± 0.016	0.500 ± 0.012	0.600 ± 0.022	0.525 ± 0.01
	BayesB	0.635 ± 0.015	0.515 ± 0.012	0.615 ± 0.025	0.531 ± 0.009
	BayesC	0.622 ± 0.016	0.508 ± 0.011	0.615 ± 0.025	0.534 ± 0.009
	deepGBLUP	*0.660* ± *0.016*	*0.544* ± *0.013*	*0.650* ± *0.023*	*0.552* ± *0.01*
1000	GBLUP	0.532 ± 0.017	0.384 ± 0.02	0.528 ± 0.018	0.424 ± 0.014
	DGBLUP	0.532 ± 0.017	0.381 ± 0.021	0.527 ± 0.018	0.424 ± 0.014
	EGBLUP	0.532 ± 0.017	0.384 ± 0.02	0.527 ± 0.018	0.423 ± 0.014
	BayesA	0.487 ± 0.018	0.361 ± 0.022	0.479 ± 0.018	0.404 ± 0.016
	BayesB	0.502 ± 0.015	0.365 ± 0.019	0.496 ± 0.019	0.405 ± 0.014
	BayesC	0.505 ± 0.015	0.365 ± 0.02	0.510 ± 0.018	0.402 ± 0.016
	deepGBLUP	*0.557* ± *0.018*	*0.432* ± *0.018*	*0.564* ± *0.019*	*0.438* ± *0.013*

Each value in the cells are means and standard errors of the predictive abilities for 10-fold tests. We highlight the best results in *italic*

#### Impact of each component

We studied the contribution of four components: (1) deep learning networks $\hat{{\textbf{b}}}_\text {deep}$, (2) additive GBLUP $\hat{{\textbf{b}}}_\text {a}$, (3) dominance GBLUP $\hat{{\textbf{b}}}_\text {d}$, (4) epistasis GBLUP $\hat{{\textbf{b}}}_\text {e}$, by designing various models with different combinations of these components. Table 5 reports the results on the Korean native cattle with 50K and a 9000 training size. The absence of a checkmark indicates that the corresponding component was excluded from the phenotype prediction.

**Table 5:** Results on the Korean native cattle data with different combinations of deepGBLUP components: (1) Deep learning networks $\hat{{\textbf{b}}}_\text {deep}$, (2) additive GBLUP $\hat{{\textbf{b}}}_\text {a}$, (3) dominance GBLUP $\hat{{\textbf{b}}}_\text {d}$, (4) epistasis GBLUP $\hat{{\textbf{b}}}_\text {e}$

Component				CWT	BF	EMA	MS
$\hat{{\textbf{b}}}_\text {deep}$	$\hat{{\textbf{b}}}_\text {a}$	$\hat{{\textbf{b}}}_\text {d}$	$\hat{{\textbf{b}}}_\text {e}$				
✓				0.746 ± 0.017	0.661 ± 0.009	0.722 ± 0.014	0.622 ± 0.011
✓	✓			0.753 ± 0.015	0.673 ± 0.009	0.744 ± 0.016	0.666 ± 0.012
✓		✓		0.748 ± 0.017	0.659 ± 0.01	0.725 ± 0.014	0.623 ± 0.011
✓			✓	0.747 ± 0.016	0.671 ± 0.009	0.734 ± 0.016	0.646 ± 0.012
✓	✓	✓		*0.755* ± *0.016*	0.672 ± 0.009	0.746 ± 0.016	0.666 ± 0.012
✓	✓		✓	0.751 ± 0.015	0.673 ± 0.009	0.744 ± 0.017	0.672 ± 0.012
✓		✓	✓	0.748 ± 0.016	0.669 ± 0.009	0.736 ± 0.016	$\underline{0.647 \pm 0.011}$
	✓	✓	✓	$\underline{0.725 \pm 0.016}$	$\underline{0.639 \pm 0.01}$	$\underline{0.722 \pm 0.019}$	0.665 ± 0.014
✓	✓	✓	✓	0.752 ± 0.016	*0.673* ± *0.009*	*0.746* ± *0.017*	*0.672* ± *0.012*

The absence of a checkmark indicates that the corresponding component is excluded from the phenotype prediction. We highlight the best results in *italic* and the worst results in underline

**Table 6:** Performance comparison of deepGBLUP with the other genomic prediction methods on the simulated data across different heritabilities and single QTL effects

Heritability	Method	QTL effect		
		a	d	e
0.5	GBLUP	0.633 ± 0.008	0.629 ± 0.008	0.613 ± 0.005
	DGBLUP	0.627 ± 0.008	0.624 ± 0.007	0.608 ± 0.005
	EGBLUP	0.630 ± 0.009	0.626 ± 0.008	0.611 ± 0.006
	BayesA	0.628 ± 0.01	0.622 ± 0.007	0.606 ± 0.006
	BayesB	0.626 ± 0.009	0.621 ± 0.008	0.602 ± 0.005
	BayesC	0.628 ± 0.009	0.625 ± 0.008	0.608 ± 0.005
	deepGBLUP	*0.641* ± *0.007*	*0.635* ± *0.007*	*0.620* ± *0.006*
0.3	GBLUP	0.588 ± 0.028	0.571 ± 0.026	0.566 ± 0.027
	DGBLUP	0.587 ± 0.029	0.571 ± 0.027	0.567 ± 0.027
	EGBLUP	0.587 ± 0.028	0.571 ± 0.026	0.565 ± 0.027
	BayesA	0.569 ± 0.027	0.552 ± 0.025	0.546 ± 0.026
	BayesB	0.583 ± 0.028	0.568 ± 0.026	0.564 ± 0.026
	BayesC	0.581 ± 0.028	0.567 ± 0.027	0.564 ± 0.027
	deepGBLUP	*0.608* ± *0.028*	*0.594* ± *0.026*	*0.589* ± *0.026*
0.1	GBLUP	0.457 ± 0.028	0.443 ± 0.023	0.433 ± 0.026
	DGBLUP	0.454 ± 0.028	0.441 ± 0.023	0.431 ± 0.026
	EGBLUP	0.462 ± 0.028	0.450 ± 0.023	0.439 ± 0.026
	BayesA	0.413 ± 0.031	0.388 ± 0.029	0.394 ± 0.033
	BayesB	0.446 ± 0.025	0.438 ± 0.025	0.415 ± 0.024
	BayesC	0.443 ± 0.029	0.439 ± 0.025	0.421 ± 0.029
	deepGBLUP	*0.542* ± *0.023*	*0.532* ± *0.019*	*0.518* ± *0.022*

Each value in the cells are means and standard errors of the predictive abilities for 10-fold tests. We highlight the best results in *italic*

In Table 5, the best result for each trait consistently contains the $\hat{{\textbf{b}}}_\text {deep}$ component. However, exclusion of $\hat{{\textbf{b}}}_\text {deep}$ led to the worst result with only one exception. These results validate that the deep learning networks based on LCL can estimate more accurate marker effects and increase model performance compared to the regular GBLUP.

**Table 7:** Performance comparison of deepGBLUP with the other genomic prediction methods on the simulated data across different heritabilities and multiple QTL effects

Heritability	Method	QTL effect			
		a + d	a + e	d + e	a + d + e
0.5	GBLUP	0.628 ± 0.009	0.614 ± 0.007	0.610 ± 0.007	0.610 ± 0.008
	DGBLUP	0.622 ± 0.009	0.609 ± 0.007	0.606 ± 0.007	0.606 ± 0.008
	EGBLUP	0.626 ± 0.01	0.612 ± 0.008	0.609 ± 0.007	0.610 ± 0.009
	BayesA	0.622 ± 0.01	0.608 ± 0.008	0.604 ± 0.007	0.606 ± 0.009
	BayesB	0.622 ± 0.009	0.607 ± 0.008	0.601 ± 0.006	0.603 ± 0.008
	BayesC	0.627 ± 0.009	0.608 ± 0.007	0.606 ± 0.007	0.607 ± 0.008
	deepGBLUP	*0.636* ± *0.009*	*0.623* ± *0.006*	*0.618* ± *0.006*	*0.620* ± 0.007
0.3	GBLUP	0.579 ± 0.025	0.572 ± 0.026	0.557 ± 0.026	0.565 ± 0.025
	DGBLUP	0.578 ± 0.026	0.573 ± 0.027	0.559 ± 0.026	0.566 ± 0.025
	EGBLUP	0.579 ± 0.026	0.571 ± 0.026	0.558 ± 0.026	0.565 ± 0.025
	BayesA	0.563 ± 0.025	0.552 ± 0.027	0.542 ± 0.025	0.549 ± 0.024
	BayesB	0.574 ± 0.026	0.563 ± 0.026	0.553 ± 0.027	0.559 ± 0.025
	BayesC	0.574 ± 0.026	0.570 ± 0.027	0.553 ± 0.026	0.562 ± 0.025
	deepGBLUP	*0.601* ± *0.026*	*0.593* ± *0.026*	*0.583* ± *0.026*	*0.585* ± *0.025*
0.1	GBLUP	0.453 ± 0.024	0.441 ± 0.027	0.427 ± 0.022	0.438 ± 0.023
	DGBLUP	0.450 ± 0.024	0.438 ± 0.027	0.425 ± 0.022	0.435 ± 0.023
	EGBLUP	0.459 ± 0.024	0.446 ± 0.026	0.435 ± 0.022	0.444 ± 0.022
	BayesA	0.408 ± 0.028	0.390 ± 0.03	0.377 ± 0.028	0.399 ± 0.027
	BayesB	0.436 ± 0.024	0.435 ± 0.028	0.417 ± 0.023	0.433 ± 0.021
	BayesC	0.446 ± 0.026	0.439 ± 0.03	0.421 ± 0.025	0.431 ± 0.023
	deepGBLUP	*0.528* ± *0.018*	*0.524* ± *0.026*	*0.513* ± *0.017*	*0.507* ± *0.018*

Each value in the cells are means and standard errors of the predictive abilities for 10-fold tests. We highlight the best results in *italic*

Furthermore, excluding the GBLUP framework from deepGBLUP results in substantial decreases in predictive abilities (Table 5). Even though the deep learning networks improve the performance of deepGBLUP, the integration of the GBLUP method is still critical to the overall model performance.

### Model performance on the simulated data

The simulated dataset was used to evaluate the performance of deepGBLUP across various heritabilities and QTL effects. As in the Korean native cattle data, we also used the validation stage for model training and the predictive ability for performance measurement. Tables 6 and 7 compare deepGBLUP with the other methods for single and multiple QTL effects. We observed that the proposed deepGBLUP achieves superior performance compared to both GBLUP and Bayesian methods for all heritabilities and QTL effects. In particular, deepGBLUP markedly outperforms the other methods in lower heritability scenarios. These results demonstrate that deepGBLUP can implement accurate genomic predictions even when the genetic variance is relatively small compared to the phenotypic variance.

## Discussion

### Deep learning for genomic prediction

Many existing studies, which use deep learning networks for genomic prediction, have relied on previous local based architectures, such as CNN or RNN [22, 23, 37]. These methods assign variant effects based on the patterns of adjacent markers. Although adjacent markers can be useful information in whole genome sequence data, they often lack inherent functional context (e.g. protein coding) in SNP array data. In other words, the adjacent SNPs with the same sequence but located in different loci should have different functional effects from each other. Therefore, these approaches are not appropriate from a genetics perspective and have shown lower prediction accuracy than the other state-of-the-art methods such as GBLUP and Bayesian methods [22, 23, 37]. In contrast, we used a locally-connected layer that can estimate distinct weight sets for adjacent SNPs located in different loci. Our results show that the LCL-based deep learning networks improved model performance from the previous methods.

In [25], Pook et al. also used an LCL-based model for genomic prediction, but their approach predicts GV directly through a sequential deep learning network, and this simplistic structure did not achieve higher performance compared to the other prediction methods. As in Tables 3 and 5, the proposed deepGBLUP also underperformed compared to the other methods, if the GBLUP framework was excluded. These results suggest that the combined use of both GBLUP and deep learning networks is crucial for improving prediction accuracy. Furthermore, Pook et al. required large-scale datasets in order to achieve comparable performance to the other methods [25]. On the contrary, the proposed deepGBLUP yielded stable performance with relatively few training data (1K).

Transformer [16] is another alternative to estimate marker effects from SNP data. It can effectively assign the effects of adjacent markers by considering their loci and patterns. However, this method demands a larger training dataset compared to the other deep learning architectures in order to achieve similar performance. For instance, in the computer vision task [17], the transformer required more than 300 M training images to outperform previous methods. This is not practical for genomic prediction due to the high cost and time consumption for animal genotyping.

### Limitations

Even though deepGBLUP has demonstrated reliable GV predictions for the Korean native cattle, there are still limitations in its flexibility for its use across various populations. In this study, we evaluated the performance of deepGBLUP using the test individuals that were in the same generation as the training individuals. Since the Korean native cattle is a relatively long-established breed, individuals in the same generation share similar genetic patterns. In other words, the training population in this study may include primitive features of the test population. To validate deepGBLUP more precisely, it needs to be evaluated by an across-breed or multi-generation test. Specifically, the performance of deepGBLUP should be measured using test individuals that belong to different breeds or generations with the training individuals.

To challenge these experimental limitations, we implemented a forward-in-time evaluation on the Korean native cattle dataset. Specifically, we constructed a validation population with 1154 individuals born in 2017, and a training population of 8846 individuals born between $2010\sim 2016$. Table 8 shows that the proposed deepGBLUP consistently outperformed the other methods for all traits, as demonstrated by the cross-validation approach.

**Table 8:** Performance comparison of deepGBLUP with the other genomic prediction methods using forward-in-time evaluation on the Korean native cattle dataset

Method	CWT	BF	EMA	MS
GBLUP	0.684	0.638	0.566	0.594
DGBLUP	0.684	0.621	0.573	0.589
EGBLUP	0.670	0.628	0.561	0.586
BayesA	0.707	0.640	0.553	0.587
BayesB	0.714	0.649	0.557	0.584
BayesC	0.711	0.628	0.574	0.599
deepGBLUP	*0.718*	*0.670*	*0.592*	*0.603*

Each value in the cells is the predictive ability. We highlight the best results in *italic*

In addition, deepGBLUP needs the genotypes of all the animals to estimate their GV. However, the common practice in animal breeding is to perform a joint GV estimation for both genotyped and non-genotyped animals. To enable more extensive applications, deepGBLUP needs to be further developed to estimate GV simultaneously for genotyped and non-genotyped animals. As a potential solution, deepGBLUP will provide an option to use a pedigree module, which approximates GV from pedigree information for non-genotyped animals.

In this study, we integrated deep learning networks with GBLUP methods and markedly increased predictive abilities from the regular GBLUP. However, deepGBLUP can also replace the GBLUP framework with other prior methods to estimate auxiliary GV as illustrated in Fig. 2. Therefore, possible future developments include integrating deepGBLUP with other existing models, such as Bayesian methods, for more accurate genomic prediction.

## Conclusions

In this paper, we introduce deepGBLUP, a novel genomic prediction algorithm for complex traits in the Korean native cattle. The main contribution of deepGBLUP is the combination of deep learning networks and a GBLUP framework in a single model. Given an input SNP data, the deep learning networks extract the effects of adjacent SNPs using locally-connected layers and subsequently use them to estimate an initial GV through fully-connected layers. The GBLUP framework estimates three types of GV (additive, dominance, and epistasis) by leveraging respective genetic relationship matrices. The proposed deepGBLUP calculates a final GV by summing all the estimated genomic values. The experimental results on the Korean native cattle data and simulated data demonstrate that the proposed deepGBLUP outperforms the previous methods, providing a reliable prediction for various traits, marker densities, training sizes, heritabilities, and QTL effects.

## Supplementary Information

**Additional file 1: Figure S1.** QTL mapping with deepGBLUP for a single QTL effect. We simulated heritability 0.5 across QTL effects including additive, dominance, and epistasis. (a) for additive QTL, (b) for dominance QTL, and (c) for epistasis QTL.** Figure S2.** QTL mapping with deepGBLUP for two QTL effects. We simulated heritability 0.5 across QTL effects including additive, dominance, and epistasis. (a) for additive+dominance QTL, (b) for additive+epistasis QTL, and (c) for dominance+epistasis QTL.** Figure S3.** QTL mapping with deepGBLUP for three QTL effects. We simulated heritability 0.5 across QTL effects including additive, dominance, and epistasis. (a) for additive+dominance+epistasis QTL.

## Data Availability

All source code and sample data in this study are freely available at https://github.com/gywns6287/deepGBLUP. Request for Genotype data can be made to Korea National Institute of Animal Science, Animal Genome & Bioinformatics Division (http://www.nias.go.kr/english/sub/boardHtml.do?boardId=depintro).
