# Supplementary material for: deepGBLUP: joint deep learning networks and GBLUP framework for accurate genomic prediction of complex traits in Korean native cattle
Source: Genet Sel Evol. 2023 Jul 31;55:56. doi: 10.1186/s12711-023-00825-y (PMC10392020; doi:10.1186/s12711-023-00825-y)
Supplement: Supplementary file 1 — Additional file 1: Figure S1. QTL mapping with deepGBLUP for a single QTL effect. We simulated heritability 0.5 across QTL effects including additive, dominance, and epistasis. (a) for additive QTL, (b) for dominance QTL, and (c) for epistasis QTL. Figure S2. QTL mapping with deepGBLUP for two QTL effects. We simulated heritability 0.5 across QTL effects including additive, dominance, and epistasis. (a) for additive+dominance QTL, (b) for additive+epistasis QTL, and (c) for dominance+epistasis QTL. Figure S3. QTL mapping with deepGBLUP for three QTL effects. We simulated heritability 0.5 across QTL effects including additive, dominance, and epistasis. (a) for additive+dominance+epistasis QTL. [file 12711_2023_825_MOESM1_ESM.pdf]

RESEARCH

Supplementary Materials for deepGBLUP: Joint deep learning and GBLUP for accurate genomic prediction of complex traits in Korean native cattle.

Hyo-Jun Lee[1], Jun Heon Lee[2], Cedric Gondro[4], Yeong Jun Koh[3*] and Seung Hwan Lee[2*]

*Correspondence: yjkoh@cnu.ac.kr; slee46@cnu.ac.kr
[3] Department of Computer Science & Engineering, Chungnam National University, 305-764, Daejeon, Korea
[2] Division of Animal and Dairy Science, Chungnam National University, 305-764, Daejeon, Korea
Full list of author information is available at the end of the article

QTL-mapping with deepGBLUP

For more comprehensive evaluation of the proposed deepGBLUP, we implemented QTL-mapping with simulation dataset. The process to construct simulation data was same with one in Method section, except that the number of QTL is 10. We presents absolute weights of the last fully-connected layer as estimated marker weights. Fig. S-1, Fig. S-2, and Fig. S-3 show the QTL mapping results. We observed that the proposed deepGBLUP properly identifies the additive, dominance, and epistasis QTLs, which can result in the superior performances of genomic prediction as in Results section.

Author details

[1] Department of Bio-AI Convergence, Chungnam National University, 305-764, Daejeon, Korea. [2] Division of Animal and Dairy Science, Chungnam National University, 305-764, Daejeon, Korea. [3] Department of Computer Science & Engineering, Chungnam National University, 305-764, Daejeon, Korea. [4] Department of Animal Science, Michigan State University, East Lansing, MI, USA.

References

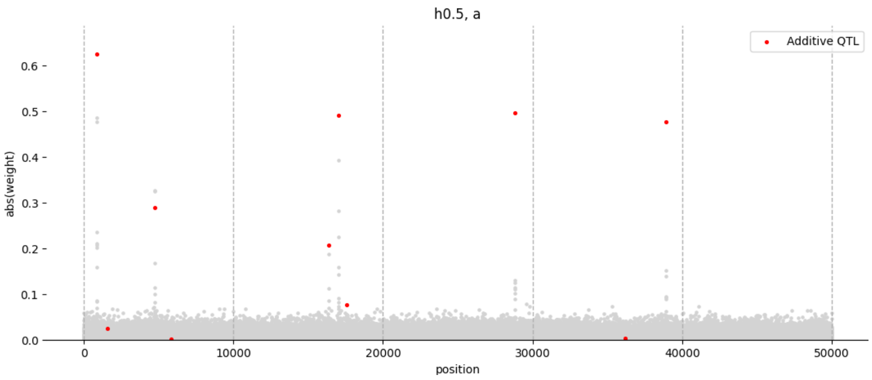

(a)

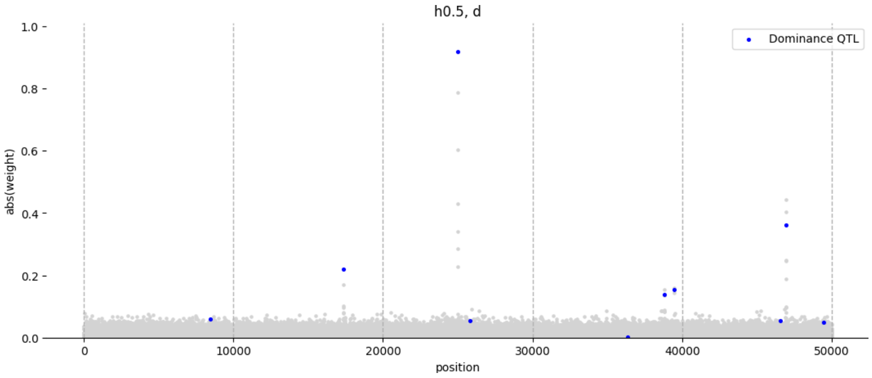

(b)

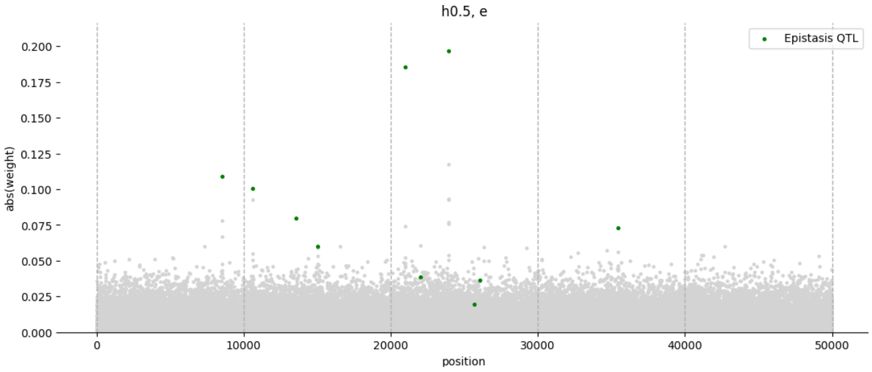

(c)

Figure S-1 QTL mapping with deepGBLUP for a single QTL effect. We simulated heritability 0.5 across QTL effects including additive, dominance, and epistasis. (a) for additive QTLs, (b) for dominance QTLs, and (c) for epistasis QTLs.

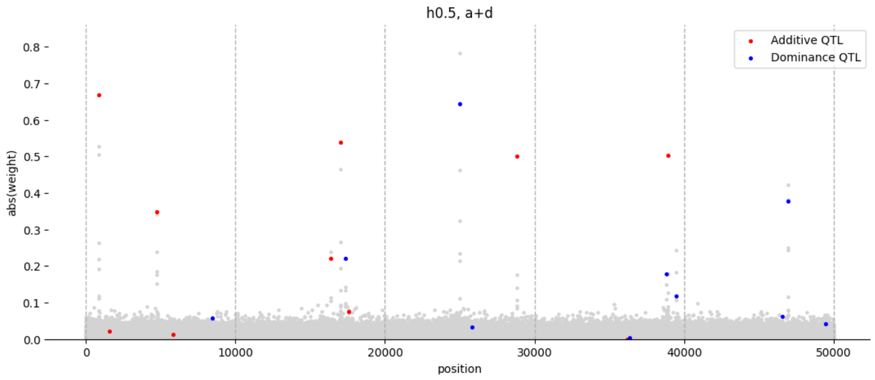

(a)

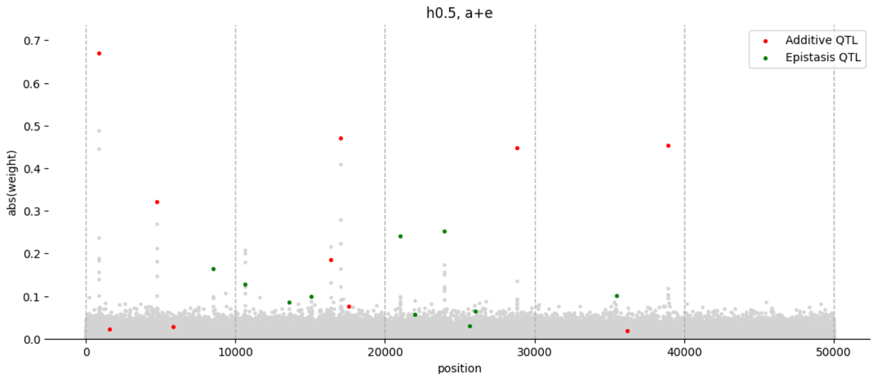

(b)

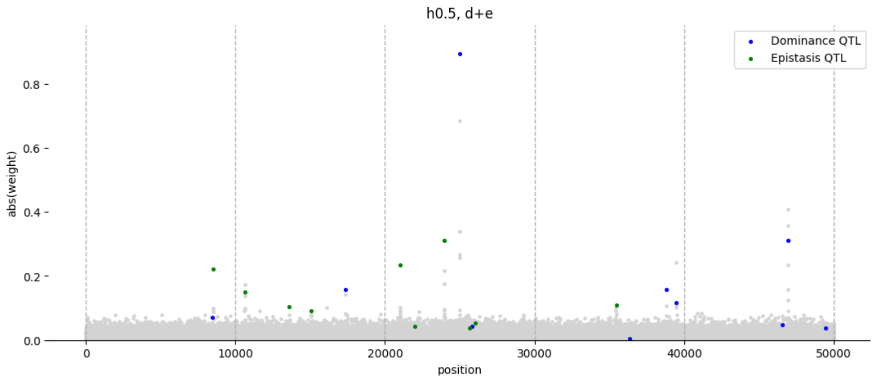

(c)

Figure S-2 QTL mapping with deepGBLUP for two QTL effects. We simulated heritability 0.5 across QTL effects including additive, dominance, and epistasis. (a) for additive+dominance QTLs, (b) for additive+epistasis QTLs, and (c) for dominance+epistasis QTLs.

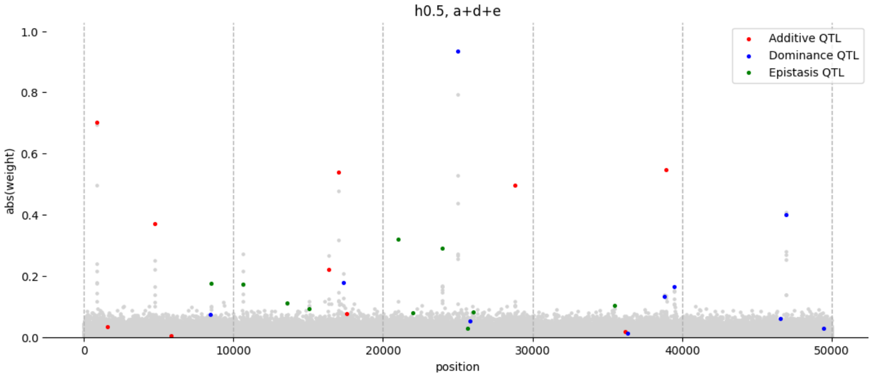

(a)

Figure S-3 QTL mapping with deepGBLUP for three QTL effects. We simulated heritability 0.5 across QTL effects including additive, dominance, and epistasis. (a) for additive+dominance+epistasis QTLs.
